# A Rare Case of Patella Rotational Dislocation and Incarceration in Femoral Condyle Fracture: A Case Report

**DOI:** 10.5704/MOJ.2203.017

**Published:** 2022-03

**Authors:** YT Chong, CW Chang

**Affiliations:** 1 Department of Orthopaedics and Traumatology, Universiti Kebangsaan Malaysia, Kuala Lumpur, Malaysia; 2 Department of Orthopaedics, Hospital Serdang, Serdang, Malaysia

**Keywords:** patella dislocation, patella incarceration

## Abstract

This is a rare case report of medial femoral condyle fracture with irreducible incarcerated patella dislocation. Following the literature search, only a few cases have been reported in the literature. In this case, the patient had undergone open reduction and screw fixation of the femoral condyle, augmented with a distal femoral locking plate (LCP). Postoperatively patient was immobilised with an above knee backslab for a month. After removing the backslab, physiotherapy was commenced to improve the range of motion and strengthening the quadriceps muscle. After 18 months of follow-up, the patient recovered well with a satisfactory bone union, no episode of patella dislocation, full weight bearing with an acceptable range of motion of his left knee about 0° to 90°. He could squat, climb stairs, and walk without any walking aid and returned to work as a food deliverer.

## Introduction

Rotational dislocation of patella with patella incarceration in fracture sites is rare in the literature. They are usually difficult to reduce by closed methods. These dislocations can be associated with osteochondral and retinacular injury, therefore open reduction is preferred^1^. Rotational patellar dislocations are rare where the patella rotates about its horizontal-vertical axis as they have usually been classified accordingly as horizontal or vertical dislocations^2^.

We report a case of a rare presentation of patella rotational dislocation and incarceration in a femoral condyle following a motor vehicle accident and how early surgical intervention has resulted in a good outcome.

## Case Report

**Fig. 1:**
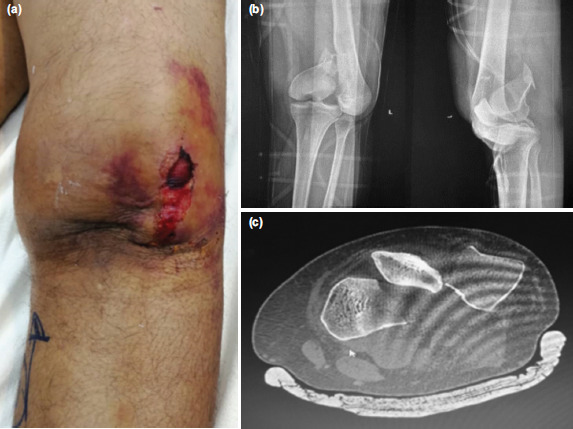
(a) Physical examination revealed a swollen left knee with a palpable gap at the anterior knee, patella cannot be felt. (b) Plain radiograph of left knee showing patella was rotated and incarcerated in medial condyle femur fracture site (c) Axial view of left knee CT, showing rotational dislocation of the patella.

A 23-year-old man with no underlying comorbidities presented with pain and swelling over his left knee following a road traffic accident. Examination showed his knee was locked in extension with massive swelling. There was no external wound but a palpable gap over the anterior aspect of the knee and was extremely tender (Fig.1a). Otherwise, the limb neurovascular status was intact. The plain radiograph showed a partial articular fracture of medial femoral condyle with patella dislocation (Fig.1b). The patella was rotated along its longitudinal axis incarcerated in the fracture site. Computed Tomography (CT) scan was ordered to assess the fracture configuration and diagnosis was confirmed. (Fig.1c).

**Fig. 2:**
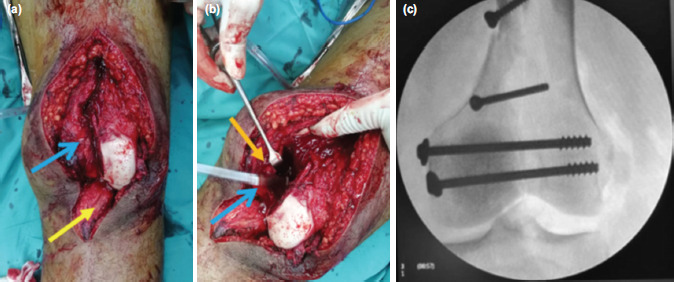
(a) Intra-operative photo showing patella incarcerated in the fracture site ( ) patella tendon ( ), (b) medial condyle of the femur was retracted ( ) to disengage the incarcerated patella ( ), (c) after disengagement of the patella, medial condyle of the femur was able to be restored and fixed with lag screws, reduction checked under image intensifier.

During surgery, we found that his left patella, along with patella tendon and quadriceps tendon that were partially torn, was incarcerated inside the fractured condyles (Fig. 2a). After disengaging the incarcerated extensor mechanism (Fig. 2b) we were able to reduce and fix the medial femoral condyle. Reduction was good after a few lag screws were placed to hold the fracture. Definite fixation is augmented with a distal femoral locking plate (LCP) used as a neutralisation principle as the fracture extending to the metaphysis of the distal femur. The reduction was acceptable, checked under image intensify guidance (Fig. 2c). It was then followed by repairs of the quadriceps and retinaculum. Post repair, the patella was found to be stable throughout the range of motion with good patella tracking.

**Fig. 3:**
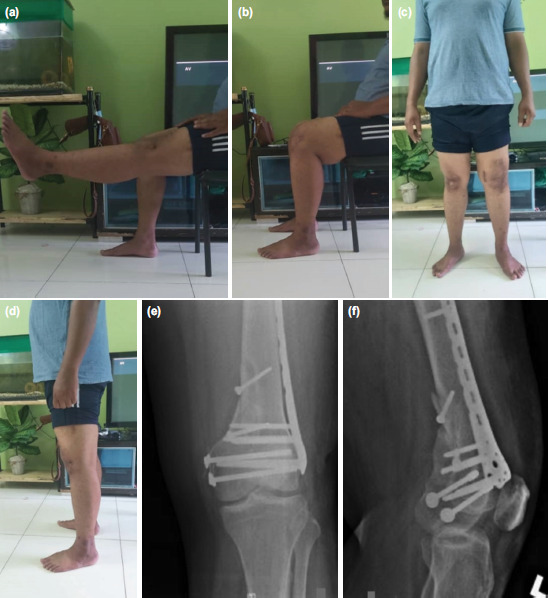
(a, b) Clinical picture showed the patient was able to flex full extension and his left knee up to 90°, (c, d) the patient was able to stand without any aids, (e, f) post-operative radiograph AP and lateral view over left distal femur showed fracture have united.

Post-operation, the left lower limb was temporarily immobilised in an above knee backslab for one month, followed by active knee range of motion exercises and quadriceps strengthening. At three months, the patient was allowed partial weight bearing. After six months, he was able to fully weight bear without support. He was able to flex his left knee up to 90° and had full extension in his knee (Fig. 3a to 3d). Radiographically, his fracture showed satisfactory union at nine months (Fig. 3e and 3f). During 18 months of follow-up, he is well, able to squat, do his prayer in a sitting position and return to his previous job where he works as a food deliverer. He has no episode of patella dislocation after the operation and able to ambulate well without aid.

## Discussion

The combination of patella rotational dislocation and incarceration in the medial condyle of femur fracture is rare with none so far in the literature search. Few articles have described incarceration of the patella in a coincident of knee fracture, they are normally associated with Hoffa fracture of the femur^2-4^.

Yazdi *et al* described a single case report of a 28-year-old with a similar mechanism of injury to our case where the patient having a Hoffa fracture and patella incarcerated in lateral femoral condyle^3^. This is perhaps the most similar to our case, the description focuses mainly on how to fix the Hoffa fracture. Whereas in this case, it is not associated with Hoffa fracture. As such, we believe that this is the first published case of its kind and is an important learning point for all clinicians involved in the management of such injuries in the future.

The main role in the management of this injury is to get an early reduction of the dislocation and relocate the patella in its pre-position. It can help reduce swelling over the knee and at the same time reduce the chances of chondral damage over the patella and femoral condyle. Early surgical management can also help in the direct repair of the medial soft tissue structures such as the medial patellofemoral ligament (MPFL), and the medial retinaculum. MPFL disruption of varying degrees have been reported in acute traumatic patellar dislocation patients, MPFL is important for preventing lateral patella dislocation^5^. MPFL is also an important structure for patella stability and the patella is an important fulcrum for the knee extension mechanism^5^.

In this case, the MPFL was completely torn with moderate disruption seen at the superomedial retinaculum and the quadriceps tendon attachment site. Surgical exposure was made using a midline incision, after disengaging the incarcerated patella from the medial side. Multiple lag screws were inserted to fix the fractured medial femoral condyle. The distal femur locking plate was used as a neutralisation plate principle to provide stable fixation to the femur as the medial condyle fracture extending up to the metaphysis of the distal femur. Ideally the plate should be placed medially, however due to risk of femoral vessels injury, we decided to place it laterally.

In conclusion, patella dislocation is an emergency condition that requires immediate reduction. In this case, closed reduction is not recommended, as it is unlikely to succeed and may even cause more chondral damage and fracture comminution. Early planned surgery aimed at gentle atraumatic reduction of the patella and achieving a good repair of surrounding soft tissue can help in early return to function and achieve a good long-term outcome.
